# Discovery of Mitophagy Inhibitors with Therapeutic Potential in Different Familial Amyotrophic Lateral Sclerosis Mutations

**DOI:** 10.3390/ijms232012676

**Published:** 2022-10-21

**Authors:** Ines Maestro, Laura R. de la Ballina, Gracia Porras, Silvia Corrochano, Eva De Lago, Anne Simonsen, Patricia Boya, Ana Martinez

**Affiliations:** 1 Centro de Investigaciones Biológicas Margarita Salas-CSIC, Ramiro de Maeztu 9, 28040 Madrid, Spain; 2 Centro de Investigación Biomédica en Red en Enfermedades Neurodegenerativas (CIBERNED), Instituto de Salud Carlos III, 28031 Madrid, Spain; 3 Department of Molecular Medicine, Institute of Basic Medical Sciences, Faculty of Medicine, University of Oslo, 0372 Oslo, Norway; 4 Centre for Cancer Cell Reprogramming, Institute of Clinical Medicine, Faculty of Medicine, University of Oslo, 0450 Oslo, Norway; 5 Hospital Clínico San Carlos, Instituto de Investigación Sanitaria San Carlos, Calle del Prof Martín Lagos s/n, 28040 Madrid, Spain; 6 Instituto Universitario de Investigación en Neuroquímica, Departamento de Bioquímica y Biología Molecular, Facultad de Medicina, Universidad Complutense, 28040 Madrid, Spain; 7 Instituto Ramón y Cajal de Investigación Sanitaria (IRYCIS), 28034 Madrid, Spain

**Keywords:** mitophagy, phenotypic assay, drug discovery, amyotrophic lateral sclerosis

## Abstract

Mitophagy is the selective degradation of mitochondria by autophagy. It promotes the turnover of mitochondria and prevents the accumulation of dysfunctional mitochondria, which can lead to cellular degeneration. Mitophagy is known to be altered in several pathological conditions, especially in neurodegenerative diseases such as amyotrophic lateral sclerosis (ALS). We recently demonstrated an increase in autophagy flux in lymphoblasts from ALS patients bearing a mutation in SOD1. Thus, the identification of mitophagy inhibitors may be a therapeutic option to recover mitochondrial homeostasis. Here, using a phenotypic mitophagy assay, we identified a new mitophagy inhibitor, the small molecule named IGS2.7 from the MBC library. Interestingly, the treatment of different cellular and in vivo models of ALS with mutations on SOD1 and *TARDBP* with this inhibitor restores autophagy to control levels. These results point mitophagy inhibitors, especially IGS2.7, to a new therapeutic approach for familial ALS patients.

## 1. Introduction

The Amyotrophic lateral sclerosis (ALS) is a devastating neurodegenerative disease that affects both upper and lower motoneurons (MNs) in the brain and in the spinal cord. Hallmarks of this pathology include altered RNA metabolism, defects in axonal transport, neuroinflammation, excitotoxicity, protein aggregation, and mitochondrial dysfunction, among others [1]. The accumulation of damaged mitochondria and misfolded proteins can both be a consequence of defects in autophagy.

Autophagy is a cellular process by which cytoplasmic content, including whole organelles, is engulfed by a double membrane organelle called an autophagosome, which fuses with lysosomes to degrade the cargo and recycle the degradation products into the cytosol. Dysfunctional autophagy stresses the cell and causes the accumulation of damaged organelles and protein aggregates, leading to several pathological conditions, including neurodegeneration [2]. Impaired mitophagy, which is the selective degradation of mitochondria by autophagy, has also been reported to be involved in several pathologies [3].

Different mitophagy pathways have been described. Pink1/Parkin (PRKN)-dependent mitophagy is the most studied one. Upon mitochondrial membrane depolarization, Pink1 anchors in the outer mitochondrial membrane where it phosphorylates PRKN, an E3 ubiquitin ligase. Once active, PRKN ubiquitinates residues from the surface of the mitochondria, which allows the recruitment of autophagy receptors. The last ones bind the mitochondria to the autophagosomes, forming mitophagosomes. The second most studied pathway is receptor-mediated mitophagy or PRKN-independent mitophagy, which relays on the ability of mitochondrial receptors, such as Bcl-2 interacting protein 3 (Bnip3) of binding directly microtubule-associated protein 1A/1B-light chain 3 (LC3) in a ubiquitin-independent way [4].

The characterization of the role of autophagy and mitophagy in ALS has been the scope of several studies to better understand this devastating disease, as well as the role of non-cell autonomous mechanisms involved in the pathophysiology, such as glial cells and neuroinflammation [5]. However, although there is increasing research focused on the alterations of autophagy/mitophagy in ALS, the results are controversial. In a mouse model with superoxide dismutase 1 (SOD1) mutation, adenine monophosphate-activated protein kinase (AMPK) activation and mTOR repression suggested autophagy induction [6]. Moreover, LC3-II protein levels were increased in MN cultures expressing a SOD1^G93A^ plasmid, as well as in the spinal cord and brainstem of SOD1^G93A^ mice [7]. However, another study demonstrated a block in autophagy and the accumulation of autophagic cargo, probably due to lysosomal defects [8]. In addition, mutations in genes related to autophagy have been described in ALS patients [9]. Thus, due to the important role of autophagy in removing defective and accumulated organelles, the enhancement of autophagy has been proposed to be beneficial, although the results are debatable.

Recently, our group found that autophagy flux was enhanced in lymphoblasts from ALS patients bearing a mutation in SOD1 compared with controls [10]. These data suggest that excessive autophagy is happening as part of the pathological process and put an emphasis on the need for good inhibitors for this pathway in order to relieve the pathological process. Although the data are controversial, the search for new autophagy/mitophagy modulators is one of the current challenges in molecular biology and medicinal chemistry [5]. Thus, with the objective of finding new inhibitors of the autophagy pathway, we took advantage of the cell line U2OS expressing a mitophagy reporter, aiming to identify compounds able to modulate both autophagy and mitophagy. From 48 compounds preselected from our in-house Medicinal and Biological Chemical (MBC) library [11], one compound, IGS2.7, showed an inhibitory effect. IGS2.7 was further characterized in order to identify its mechanism of action. Finally, its therapeutic applicability as a mitophagy and autophagy modulator for ALS was studied in several familial models of ALS, both in cellular models and different spinal cord tissues from animal models.

## 2. Results

### 2.1. Phenotypic Screen to Identify Mitophagy Inhibitors

**Figure 1:**
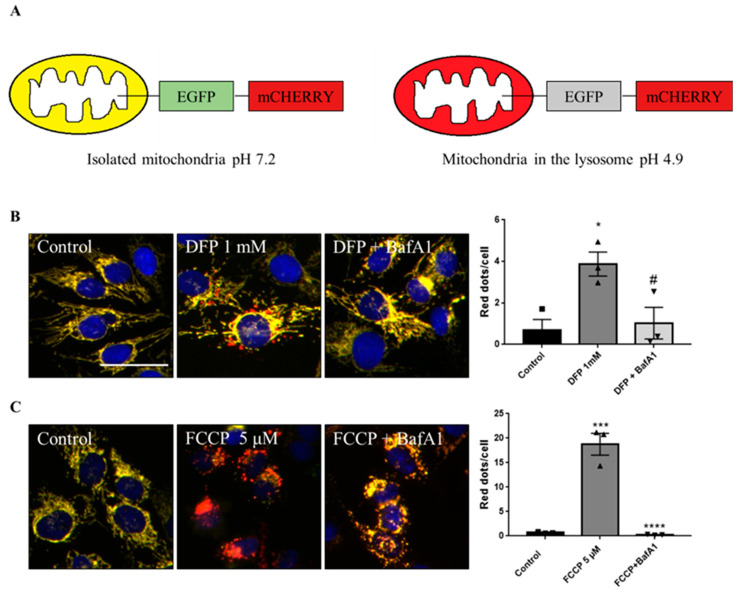
Description of the iMLS reporter and assay setup. (**A**) Mitophagy reporter description. (**B**) Representation and quantification of mitophagy modulations in U2OS-iMLS cells treated with DFP for 24 h. BafA1 was added for the last 2 h. (**C**) Representation and quantification of mitophagy modulations in U2OS-iMLS-PRKN cells treated with FCCP for 24 h. BafA1 was added for the last 2 h. Data represent the mean ± SEM of three experiments (significance was determined by one-way ANOVA followed by Dunnett’s multiple comparisons test where * *p* < 0.05 and *** *p* < 0.001 compared with control and # *p* < 0.05 and **** *p* < 0.0001 compared with DFP or FCCP, respectively). Scale bar = 50 µm.

To discover mitophagy inhibitors, we used the U2OS cell line with doxycycline-inducible expression of the double-tagged NIPSNAP1 internal mitochondrial localization signal (iMLS, NIPSNAP1^1–53^-EGFP-mCherrry), previously described as a mitophagy reporter [12]. Similar to other tandem reporters, the phenotypic assay is based on pH changes. When mitochondria are in the cytosol at pH 7.2, the reporter is seen in yellow, a combination of the green and red fluorophores. When mitochondria are inside the lysosome, the lower pH (~4.9) quenches the green fluorophore, and the reporter is seen only in red (Figure 1A). Thus, this reporter allows the discrimination between yellow mitochondrial network and red-only structures, representing mitochondria in lysosomes [13,14].

Because U2OS cells express low levels of PRKN, we also used U2OS-iMLS cells transduced with a lentiviral particle expressing PRKN (U2OS-iMLS-PRKN) [14]. Thus, this combination of cell lines allows us to search for inhibitors of both PRKN-independent and PRKN-dependent mitophagy. To set up the screening, we first tested the validity of the system by measuring the response to several positive and negative controls. The negative control was DMSO 0.1%, which was used as the vehicle in which the compounds were dissolved. As positive controls, U2OS-iMLS were treated with deferiprone (DFP), an iron chelator know to induce receptor-mediated mitophagy [15] (Figure 1B), while U2OS-iMLS-PRKN cells were treated with carbonyl cyanide-p-trifluoromethoxyphenylhydrazone (FCCP), a mitochondrial uncoupler that leads to a massive turnover of mitochondria via PRKN recruitment [16] (Figure 1C). The V-ATPase inhibitor bafilomycin A1 (BafA1) was added for the last 2 h, which increases the pH, restores the EGFP signal in the acidic compartments, and blocks the fusion between autophagosomes and lysosomes [17]. As expected, DFP and FCCP significantly increased mitophagy, which was completely blocked when BafA1 was added (Figure 1B,C).

**Figure 2:**
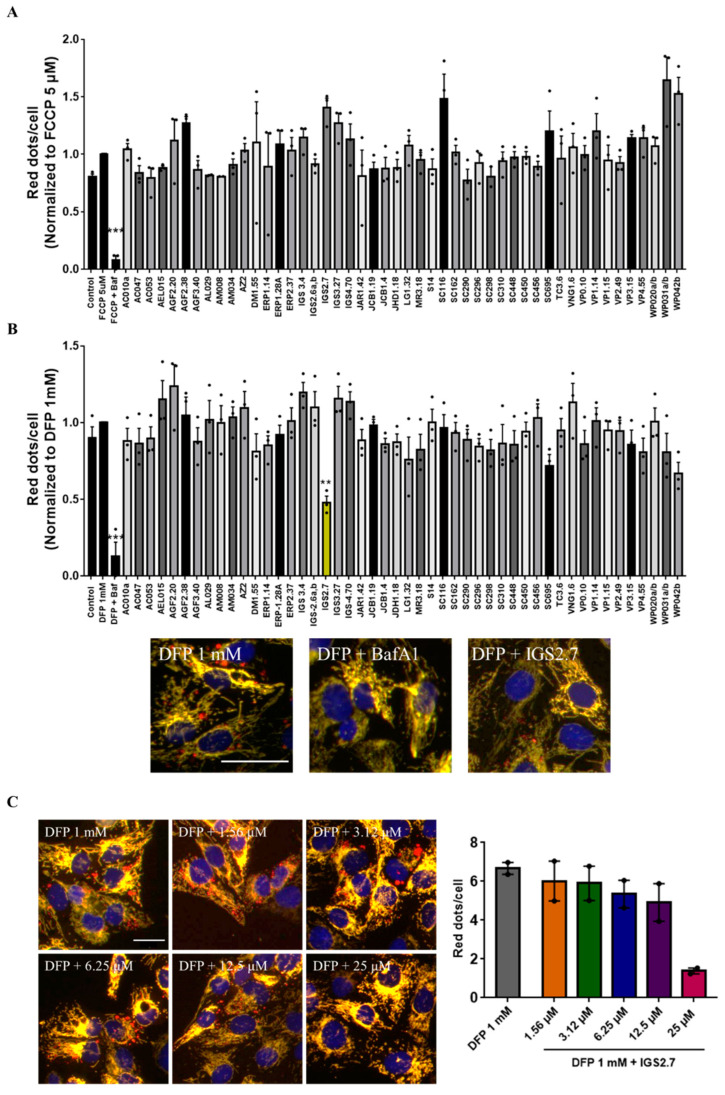
IGS2.7 inhibited DFP-induced mitophagy in U2OS-iMLS cells. (**A**) U2OS-iMLS-PRKN and (**B**) U2OS-iMLS cells were co-treated with 5 µM FCCP or 1 mM DFP, respectively, and compounds preselected from the MBC library at 25 μM for 24 h prior to high content imaging and image analysis. Data represent the mean ± SEM of three independent experiments. Only IGS2.7, in yellow, reduced DFP-induced mitophagy in U2OS-iMLS cells (significance was determined by one-way ANOVA followed by Dunnett’s multiple comparison test to control, where *** *p*  <  0.001, ** *p* < 0.005). Scale bar = 50 µm. (**C**) Representation and quantification of mitophagy in U2OS-iMLS cells treated with DFP and increasing doses of IGS2.7. Data represent mean ± SEM of two independent experiments (significance was determined by one-way ANOVA followed by Dunnett’s multiple comparison test to control, where ** *p* < 0.01). Scale bar = 30 µm.

We next treated both cell lines with compounds from the MBC library in the absence or presence of the mitophagy inducers. The selection of the compounds was based on the chemical structure and diversity of biological activities. All these compounds were evaluated at the same fixed dose. As shown in Figure 2A, none of the compounds were able to reduce the increase in mitophagy caused by FCCP. It is important to note that FCCP produced massive mitophagy and the mitochondrial network was almost completely lost (Figure 1C). Although BafA1 was able to block the pathway, none of the compounds were potent enough to inhibit it.

When co-treating U2OS-iMLS with the compounds and DFP as the mitophagy inducer, we found that besides the effect of the control treatment with BafA1, a compound from the MBC library, named IGS2.7 and marked in yellow in the graph, reduced DFP-induced mitophagy more than 50% (Figure 2B).

Thus, we continued characterizing IGS2.7 as a new mitophagy inhibitor. We first performed an analysis of the dose–response relationship of IGS2.7 in U2OS-iMLS cells. Cells were treated with 1 mM DFP and with increasing concentrations of IGS2.7 from 1.56 to 25 µM (Figure 2C). As previously seen, DFP induced mitophagy and the co-treatment with IGS2.7 reduced mitophagy in a dose-dependent manner, being almost completely blocked at the highest concentration of 25 µM. Thus, we confirmed that 25 µM is the concentration at which IGS2.7 inhibited DFP-induced mitophagy.

**Figure 3:**
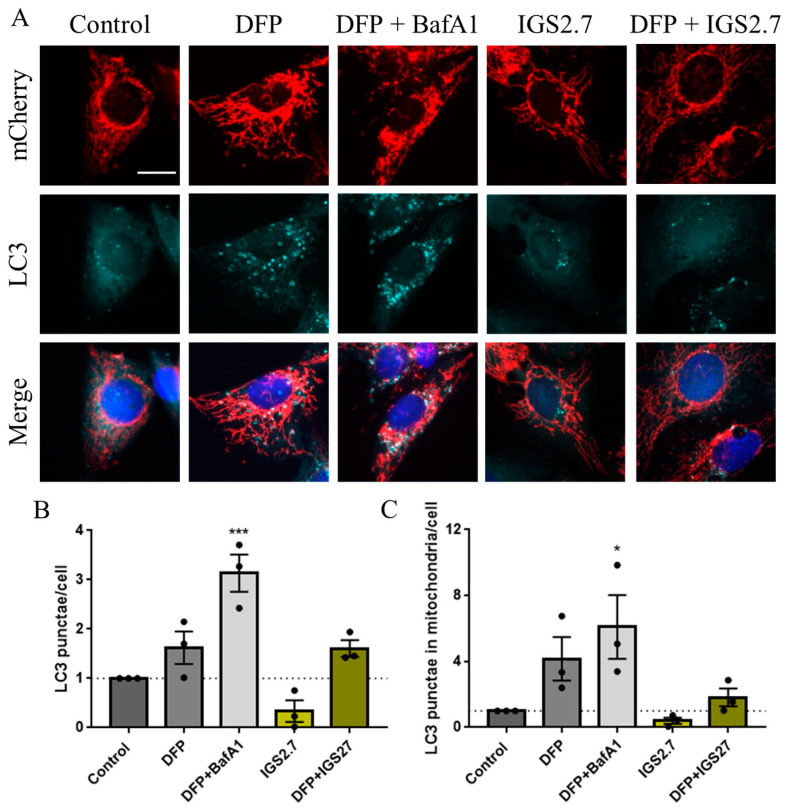
IGS2.7 reduced LC3 punctae and LC3 co-localization with mitochondria in U2OS-iMLS cells. (**A**) Immunostaining for LC3 and quantification of (**B**) LC3 punctae and (**C**) LC3 punctae in mitochondria after the treatment with IGS2.7 25 µM, DFP 1 mM for 24 h and BafA1 for the last 2 h. Data represent the mean ± SEM of three independent experiments (significance was determined by one-way ANOVA followed by Dunnett’s multiple comparison test to control, where *** *p*  <  0.001, * *p* < 0.05): Scale bar = 20 µm.

Next, we explored autophagy itself measuring the accumulation of LC3, as a marker of autophagosomes, in U2OS-iMLS treated with different conditions (Figure 3A,B). Results showed a slight increase in LC3 punctae in cells treated with DFP, as it promotes the recruitment of mitochondria by autophagosomes due to mitophagy induction. This effect was even higher upon the co-treatment with BafA1, which blocks the final step of the pathway. However, the compound IGS2.7 trended to decrease LC3 punctae in cells, which can denote a drop in autophagosome formation.

Taking advantage of the mitophagy reporter, we measured the co-localization between mitochondria, marked by the mCherry fluorophore of the iMLS reporter, and LC3 (Figure 3A,C). Similarly, DFP induced an increase in mitochondria co-localizing with LC3, which was boosted upon BafA1 treatment. This result is in agreement with the role of BafA1 as a blocker of the final step of the pathway, which promotes the accumulation of mitochondria in LC3-enriched autophagosomes. IGS2.7 slightly reduced this co-localization trending also to reduce the co-localization obtained with DFP. These results suggest that IGS2.7 may inhibit both autophagy and mitophagy pathways differently as BafA1 does, due to a mechanism of action involved in the initial steps of these processes.

### 2.2. The Ability of IGS2.7 to Block Basal Mitophagy Is Cell Type Dependent

**Figure 4:**
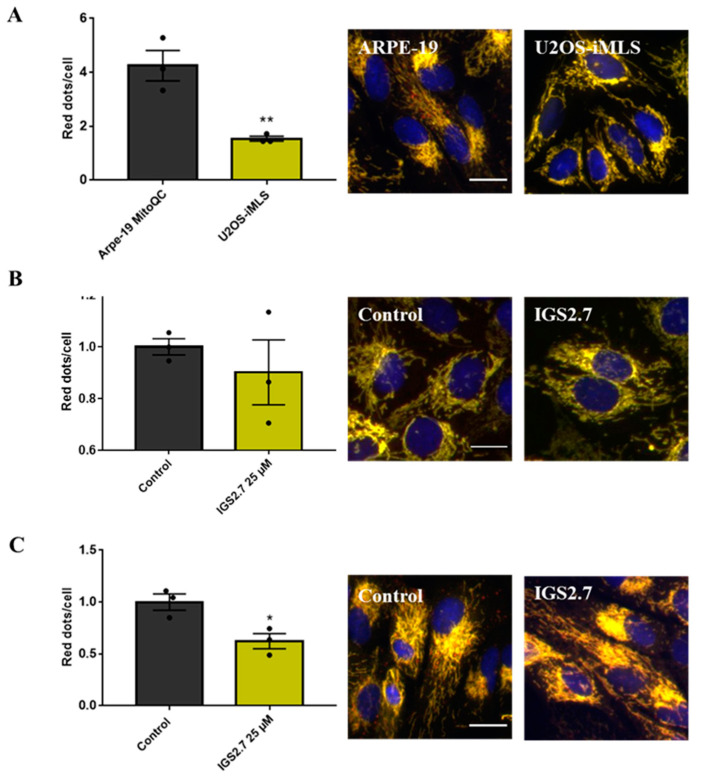
Basal mitophagy in ARPE-9 MitoQC and U2OS-iMLS cells and its modulation by IGS2.7. Representation and quantification of mitophagy in (**A**) ARPE-19 MitoQC and U2OS-iMLS cells, (**B**) U2OS-iMLS cells, and (**C**) ARPE-19 MitoQC cells treated with control and 25 μM IGS2.7 for 24 h. Data represent the mean ± SEM of three independent experiments. Data from (**B**,**C**) were normalized to control (significance was determined by unpaired two tailed *t*-test, where ** *p* < 0.01 indicates a significant difference between samples and * *p* < 0.05 indicates a significant difference from control). Scale bar = 20 µm.

We next wanted to study whether IGS2.7 was also able to block basal mitophagy. We used two different cell lines: ARPE-19 MitoQC and U2OS-iMLS cells. ARPE-19 MitoQC is a human retinal pigment epithelial cell line expressing the mitophagy reporter mCherry-GFP to the OMM protein FIS1 (known as MitoQC) [13]. Basal mitophagy levels in both cell lines were different; the level was enhanced in ARPE-19 cells (Figure 4A). No differences between the control and IGS2.7 treated cells were observed when U2OS-iMLS cells were treated with the inhibitor IGS2.7 at 25 μM for 24 h (Figure 4B), as this cell line shows low basal mitophagy. However, the number of red-only punctae decreased almost 40% compared with the control when the ARPE-19 MitoQC cells were treated with the compound at 25 µM (Figure 4C). This indicated that IGS2.7 can inhibit basal mitophagy in cells with high levels of basal mitophagy. Taken together, we conclude that IGS2.7 is able to block DFP-induced PRKN-independent mitophagy and basal mitophagy, but has no effect on PRKN-dependent FCCP-induced mitophagy.

We next used the ARPE-19 cell line for the subsequent studies.

### 2.3. Potential Mechanism of Action of IGS2.7 in Mitophagy Modulation

#### 2.3.1. IGS2.7 As a Protein Casein Kinase 1 (CK1) Inhibitor

IGS2.7 was synthesized in a medicinal chemical program to target the casein kinase 1 (CK1) [18]. CK1 is one of the kinases responsible for the phosphorylation in vivo of TAR DNA-binding protein 43 (TDP-43), encoded by *TARDBP*, an event that is very common in ALS [19]. Thus, we hypothesized that the CK1 inhibition can be a potential mechanism of action through which IGS2.7 is reducing mitophagy. However, other CK1 inhibitors (IGS3.4, IGS3.27) from the same structural family and with similar potency were included in the compound screening (Table 1), and none of them reduced mitophagy in the cell lines tested (Figure 1). Thus, we discarded CK1 inhibition as the molecular mechanism by which IGS2.7 inhibited mitophagy.

#### 2.3.2. IGS2.7 as an Unc-51 such as Kinase-1 (ULK1) Inhibitor

Because IGS2.7 is an ATP-competitive inhibitor, it has the potential to modulate other kinases. So, the selectivity of IGS2.7 at 10 µM was determined against a panel of 456 protein kinases [18]. Interestingly, besides inhibiting CK1 close to 100%, IGS2.7 inhibited the activity of ULK1 by almost 60% [18]. However, we evaluated IGS3.27 and IGS3.4 from Table 1 in a kinase assay against ULK1, and none of them inhibited its kinase activity (Table 1). Thus, ULK1 can be the specific target by which IGS2.7 modulated mitophagy and probably autophagy.

ULK1 is a kinase implicated in autophagy initiation by the ULK1 complex, which triggers the recruitment of the VPS34 complex, and subsequently produces phosphatidylinositol 3-phosphate (PI3P) needed to form the autophagosome [20]. As Figure 3 showed, IGS2.7 was not acting at the end of the pathway, ULK1 inhibition emerged as a plausible situation.

**Figure 5:**
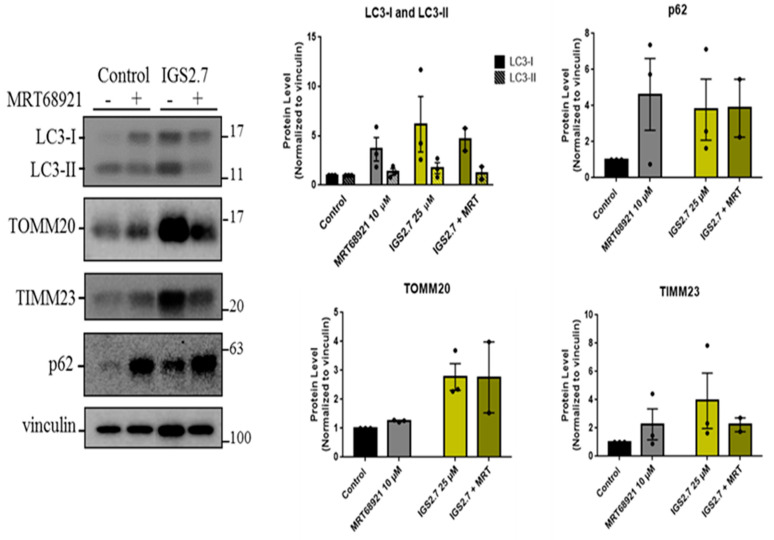
IGS2.7 acted such as the ULK1 inhibitor, MRT68921. Western blot and quantification of LC3-I, LC3-II, p62, TOMM20, and TIMM23. ARPE-19 MitoQC cells were treated with 10 µM MRT68921 and/or 25 µM IGS2.7 for 24 h. Data represent the mean ± SEM of three experiments and were normalized to control.

In order to explore if IGS2.7 modulated these pathways via ULK1, we compared it with MRT68921, an ULK1 inhibitor commonly used to block autophagy, whose potency to inhibit mitophagy has been already tested in a similar phenotypic assay [14]. As ARPE-19 MitoQC cells showed high basal mitophagy, cells were treated with those two compounds without any enhancers for 24 h, followed by Western blot analysis of the autophagy markers LC3 and p62, as well as mitochondrial inner (TIMM23) and outer (TOMM20) membrane proteins (Figure 5). Our data showed the accumulation of LC3-I upon IGS2.7 or MRT68921 treatment, which can denote a block in LC3-II lipidation and a block in the initial steps of the pathway. The autophagy substrate p62 was also accumulated with both IGS2.7 and MRT68921, which supports the idea that they both block the induction step of autophagy. Moreover, TIMM23 and TOMM20 were also accumulated when cells were treated with the inhibitor IGS2.7. This increase in mitochondrial mass is in agreement with the results from the phenotypic assay, indicating that IGS2.7 blocks mitophagy.

These results along with Figure 3 and Table 1 strengthened our hypothesis of IGS2.7 acting as an initial phase blocker that hindered the recruitment of LC3, thus inhibiting autophagy and mitophagy.

### 2.4. Therapeutic Potential of IGS2.7 in ALS Models as Autophagy/Mitophagy Inhibitor

Lastly, we explored the therapeutic potential of IGS2.7 as an autophagy/mitophagy inhibitor. Previous data from our lab demonstrated the efficacy of IGS2.7 to restore the homeostasis of *TARDBP* in lymphoblasts from ALS patients and in the TDP-43^A315T^ mouse model through CK1 inhibition [21]. Thus, we proceeded to study autophagy and mitophagy in these models with and without treatment with IGS2.7 in order to add a new therapeutic value to the compound.

#### 2.4.1. Cellular and Animal SOD1 Model of ALS

Previous work from our laboratory in lymphoblasts derived from familial SOD1-ALS patients demonstrated an increase in the autophagy flux in SOD1-ALS samples compared with control samples [10]. Because IGS2.7 inhibited DFP-induced mitophagy, we proceeded to study the therapeutic potential of IGS2.7 in modulating autophagy in different ALS models. For that, we decreased the concentration of the hit from 25 to 5 μM, as it was the concentration known to decrease the hyper-phosphorylation of TDP-43 in these cell lines.

Although lymphoblasts do not belong to the central nervous system (CNS), they show pathological hallmarks described in MN from patients, such as TDP-43 hyperphosphorylation and the presence of the protein in the cytosol [22]. Thus, samples from controls and patients bearing mutations in SOD1 were treated with IGS2.7 in the absence or presence of the lysosomal inhibitor hydroxychloroquine (HCQ). Autophagy flux was assessed by measuring the ration between LC3-II intensity with and without HCQ.

**Figure 6:**
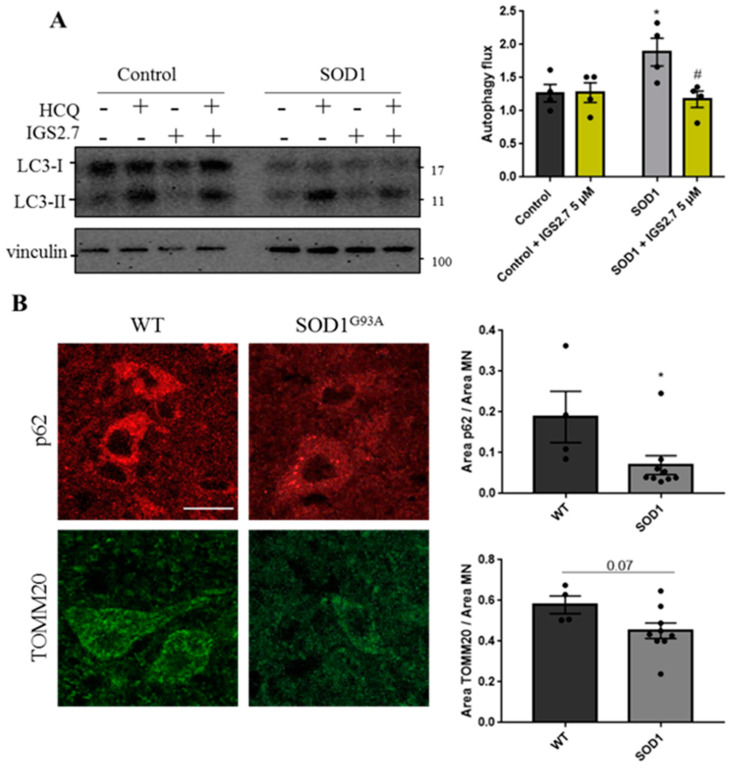
IGS2.7 reduced the autophagy flux in cellular and animal SOD1 models of ALS. (**A**) Lymphoblasts were incubated in the presence and absence of 5 µM IGS2.7 for 24 h. Hydroxychloroquine (HCQ) was added during the last 3 h when indicated. Each point represents the mean of four independent experiments. Data represent the mean ± SEM of four controls and SOD1 lymphoblastic cell lines (significance was determined by one-way ANOVA followed by Dunnett’s multiple comparisons test, where * *p*  <  0.05 vs. control and # *p* < 0.05 vs. SOD1-ALS). (**B**) Histological samples of the anterior horn of the spinal cord of SOD1^G93A^ and wild-type mice. Tissues were immunostained for p62 and TOMM20 and quantified per MN. Data represent mean ± SEM of four different control and nine transgenic mice (significance was determined by unpaired two tailed *t*-test, where * *p* < 0.05 indicates a significant difference from control). Scale bar = 20 µm.

While IGS2.7 had no effect on the autophagy flux in the control lymphoblast cells, it significantly decreased the autophagy flux in the patient samples, restoring autophagy levels to those found in healthy individuals (Figure 6A).

Because SOD1 was described as the first gene known to be mutated in ALS, several mice models have been developed. The most used mouse model is SOD1^G93A^, which better recapitulates the human pathology [23]. Here, we studied autophagy levels in this model compared with wild-type animals. We performed an immunostaining of autophagy and mitochondrial proteins in the ventral horn of spinal cords where the MN are localized (Figure 6B). Because the animals were not treated with lysosomal inhibitors, the autophagy flux could not be measured, but we used p62 levels as a surrogate marker for autophagy and TOMM20 to measure mitochondrial mass. Our data show that there is a decrease in the levels of the p62 protein per MN in SOD1^G93A^ compared with WT mice. p62 is incorporated in the autophagosome and degraded in the autolysosome, so it is considered a surrogate for the autophagy flux [24]. Thus, our data suggest an increase in autophagy in SOD1^G93A^ MNs, similar to the findings from SOD1 lymphoblasts. Moreover, the amount of mitochondrial mass measured by TOMM20 also decreased in the SOD1^G93A^ in comparison with WT animals. This can also denote an induction of mitophagy in SOD1^G93A^ animals.

#### 2.4.2. Cellular and Animal TARDBP Model of ALS

**Figure 7:**
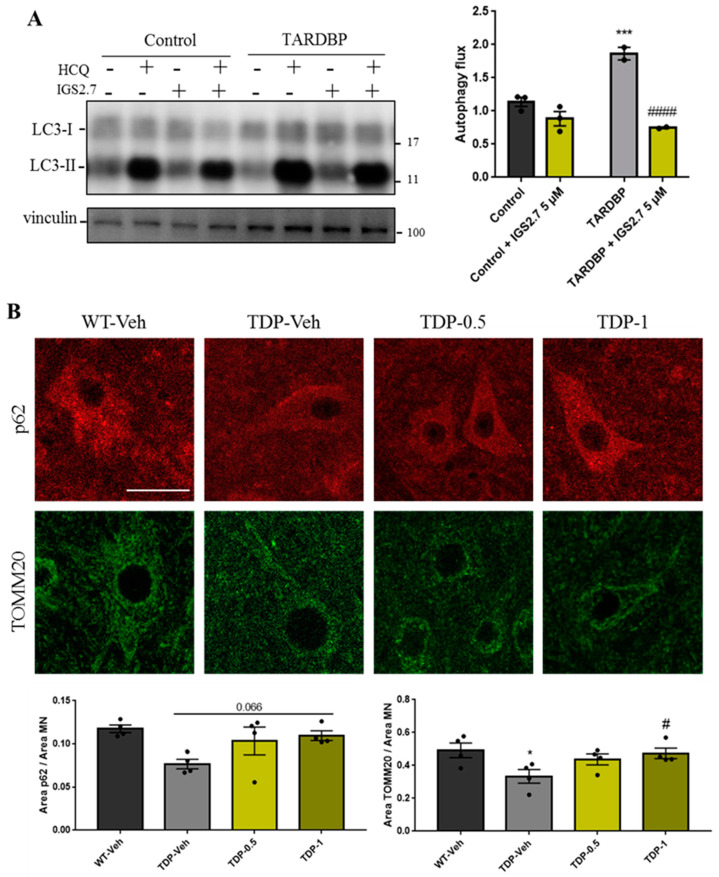
IGS2.7 restored the autophagy flux in cellular and animal *TARDBP* models of ALS. (**A**) Lymphoblasts were incubated in the presence and absence of 5 µM IGS2.7 for 24 h. Hydroxychloroquine (HCQ) was added during the last 3 h when indicated. Each point represents the mean of three independent experiments. Data represent the mean ± SEM of three controls and four TARDBP lymphoblastic cell lines (significance was determined by one-way ANOVA followed by Dunnett’s multiple comparisons test, where *** *p*  <  0.001 vs. control and #### *p* < 0.001 vs. SOD1-ALS). (**B**) Histological samples of the anterior horn of the spinal cord of TDP-43 mice and wild-type controls. Tissues were immunostained for p62 and TOMM20 and quantified per MN. Data represent mean ± SEM of four different animals (significance was determined by one-way ANOVA followed by Dunnett’s multiple comparison test, where * *p*  <  0.05 compared with WT and # *p*  <  0.05 compared with TDP-Veh). Scale bar = 30 µm.

Lastly, autophagy modulation was determined in lymphoblasts from other types of familial ALS patients. We compared the autophagy flux from lymphoblasts from patients with a mutation in *TARDBP* with control samples. Our data show that autophagy increased in *TARDBP*-ALS samples (Figure 7A) similar to SOD1-samples. In addition, the treatment with the inhibitor IGS2.7 again reduced this autophagy enhancement to the control level, thus proving the efficacy of the compound to lower high autophagy in two familial and cellular models of ALS.

Finally, we studied mitophagy and autophagy modulation in the TDP-43^A315T^ transgenic mouse model of ALS [25]. Previous data from the lab indicated a decrease in the phosphorylation of TDP-43 in combination with MN preservation and a decrease in glial reactivity after the treatment with IGS2.7 [21]. However, the modulation of autophagy and mitophagy by IGS2.7 in this mouse model has not been studied and would expand the therapeutic potential of the hit. To this end, TDP-43^A315T^ transgenic mice were treated daily with IGS2.7 at two concentrations (0.5 and 1 mg/kg) or the vehicle from postnatal day 65 to 95. Doses were selected based on previously known IGS2.7 in vivo pharmacokinetic parameters [26] and our previous experience in that animal model [21]. Wild-type animals were only treated with the vehicle. Then, we performed the immunostaining of the autophagy substrate p62 and the mitochondrial protein TOMM20.

Analysis of p62 per MN revealed a decrease in the protein in the transgenic mice compared with the levels found in control animals. Treatment with IGS2.7 trends to an increase in the p62 protein level in a dose-dependent manner; almost reaching the wild-type levels (Figure 7B). This indicates that similar to SOD1- and *TARDBP*-ALS lymphoblasts, autophagy is increased in this mice model and the treatment with IGS2.7 restored autophagy to control levels. TOMM20 staining revealed a decrease in the mitochondrial mass in the transgenic mice compared with control. The treatment with IGS2.7 restored the mitochondrial protein level to the control at the highest dose (1 mg/kg) (Figure 7B).

## 3. Discussion

Targeting autophagy and mitophagy in ALS is gaining interest in the medicinal chemistry field due to the important role of these degradative pathways in cellular homeostasis [5]. In neurodegeneration, protein aggregates and defective mitochondria accumulation encourage scientists to look for autophagy inducers to eliminate harmful cargo [27]. However, because autophagy must be maintained at healthy levels to remove defective organelles, an excessive removal of those organelles is also detrimental. In fact, autophagy induction has been reported to be controversial in ALS and depends not only on the autophagy enhancer but also on the stage of the disease [28].

We previously showed that the autophagy flux was increased in lymphoblasts from ALS patients with mutations in SOD1 in comparison with control samples [10]. This, in addition to other works in which the inhibition of the autophagy pathway improved the phenotype caused by excessive autophagy [29], encouraged us to start a drug discovery program for mitophagy inhibitors. In this study, we took advantage of the good qualities of U2OS-iMLS cells in microscopy to screen in a phenotypic assay 48 compounds from our in-house MBC library synthesized in different medical chemistry programs, preselected based on chemical diversity. Previous work from us and other groups already validated the use of this cell line to find mitophagy modulators [14,30]. In this work, the compound IGS2.7 significantly inhibited DFP-induced mitophagy in U2OS cells expressing a matrix-localized mitophagy reporter. Interestingly, U2OS cells have low levels of basal mitophagy, which was not altered by IGS2.7. All these data may indicate a potential inhibitory effect of the compound only when mitophagy levels are high enough to be modulated. This effect has been confirmed using ARPE-19 MitoQC cells, which display higher levels of basal mitophagy than those found in U2O2 cells. Treatment of ARPE-19 cells with IGS2.7 reduced basal mitophagy levels by almost half.

CK1, a protein kinase targeted by IGS2.7, has been extensively studied in ALS, and its role in TDP-43 phosphorylation is well-known [31]. However, recent data suggest a function of CK1 in autophagy. In 2020, it was reported that CK1d is essential for autophagy and its inhibition suppresses the pathway [32]. However, we tested other CK1 inhibitors with a similar structure and potency as IGS2.7, which did not inhibit mitophagy, suggesting that IGS2.7 can modulate another autophagy-related target. In fact, from a panel of 456 kinases, besides CK1, the only kinase modulated by IGS2.7 at a fixed concentration of 10 µM was ULK1, an autophagy-related kinase [18]. Western blot analysis revealed that IGS2.7 had a similar effect on autophagy proteins as MRT68921, a known ULK1 inhibitor. Both compounds caused an accumulation of the autophagy receptor p62 and the non-lipidated form of LC3 (LC3-I), as well as mitochondrial proteins. In addition, the compound acted in a different way as BafA1, a lysosomal blocker, which accumulated mitochondria in LC3-marked autophagosomes [33]. However, IGS2.7 may decrease that accumulation, probably by their role in the early phases of autophagy. All these data support the hypothesis of IGS2.7 as a mitophagy/autophagy modulator by ULK1 inhibition.

The potential therapeutic applicability of IGS2.7 as a CK1 inhibitor has been studied in depth in our group. It not only reduced pathological phospho-TDP-43 in lymphoblasts from patients but also modulated the disease in TDP-43^A315T^ mice [21], as it has been described to penetrate CNS [27]. With the objective of adding a new therapeutic value, autophagy modulation was studied in different models of familial ALS. In order to follow the same layout as the previous work and to know the efficacy of IGS2.7 in ALS treatment, lymphoblasts were treated with the compound at 5 µM, which is the concentration known to reduce TDP-43 phosphorylation. Interestingly, this low concentration was enough to decrease the excessive autophagy flux found in SOD1 and *TARDBP* lymphoblasts to levels similar to those of the control cells. In the in vivo model, the TDP-43^A315T^ transgenic mice were treated at 1 mg/kg similar to the aforementioned work and 0.5 mg/kg, which seems to restore autophagy and preserve the mitochondrial mass in a dose-dependent manner. The increase in autophagy and mitophagy in the ALS models agrees with other reports showing enhanced glycolysis and lactate production in ALS [10]. Moreover, a glycolytic shift can be promoted by enhanced mitophagy in different situations [34,35]. Mitophagy levels differ from tissues, probably depending on their metabolic demands [36]. In addition, the expression of a mitophagy-related protein, such as PRKN, is also tissue-dependent, what can differentiate from PRKN-dependent or independent mechanisms. In fact, PRKN levels are not detected in human spinal cords [37] and the recruitment of endogenous PRKN to mitochondria in neuronal cell lines is barely detected [38]. Thus, this may indicate that the enhanced mitophagy found in the MN of the spinal cords of the ALS animal models should be PRKN-independent [5]. So, the treatment of these models with IGS2.7, a receptor-mediated mitophagy inhibitor, was appropriate.

All these data may reveal the state of autophagy and mitophagy in ALS and can boost the discovery of new mitophagy modulators with a therapeutic effect in this disease. However, ALS is a complex disease in which different signaling pathways and physiological routes are altered. So, targeting only one of those detrimental pathways is not enough. What makes IGS2.7 a key molecule in the treatment of ALS is its pleiotropic effect, as it not only modulates mitophagy, but also has an important role in TDP-43 hyperphosphorylation and motor neuron preservation.

In conclusion, we identified IGS2.7 as a small molecule able to inhibit mitophagy. Importantly, its activity was only observed when mitophagy was increased, then IGS2.7 treatment is able to restore to normal levels, which is essential in therapeutics. Although CK1 inhibition may be the route by which mitophagy is modulated, our data point to simultaneous ULK1 modulation as a new target. Finally, its potency as a treatment for ALS was evaluated, and its modulation of autophagy to control levels in different models of familial ALS was demonstrated. This, in addition to its known activity in TDP-43 phosphorylation and MN preservation, makes IGS2.7 a pleiotropic drug able to modulate different pathways altered in ALS.

## 4. Materials and Methods

### 4.1. Cell Culture

U2OS-iMLS and U2OS-iMLS-PRKN FlpIn TRex cells [12] were cultured with DMEM with high glucose and glutamine and supplemented with 10% FBS and 1% Pen-Strep at 37 °C and 5% CO_2_. Cells were selected with 100 µg/mL hygromycin and 5 µg/mL blasticidin (Thermo Fisher Scientific, R21001, Waltham, MA, USA). Additional 2 µg/mL puromycin (Sigma Aldrich, P7255, St. Louis, MO, USA) were used for U2OS-iMLS-PRKN cells. To induce the expression of the mitophagy reporter, 500 µg/mL doxycycline (Clontech, 631311, Mount View, CA, USA) was added for the last 24 h. These cells were obtained from Prof. Anne Simonsen Lab (Faculty of Medicine, Institute of Basic Medical Sciences and Centre for Cancer Cell Reprogramming, Institute of Clinical Medicine, University of Oslo, Norway).

ARPE-19 MitoQC cells were maintained in DMEM:F12 (1:1) supplemented with 15% de FBS, 1% glutamine 2 mM, and 1% de pen-strep (0.5 mg/ml) at 37 °C and 5% CO_2_. Cells were selected with 800 μg/mL hygromycin. This cell line was obtained from Dr. Ian Ganley Lab (School of Life Sciences, University of Dundee, Scotland) [13].

Peripheral blood samples were obtained from four control subjects, four sporadic (sALS) patients, four ALS patients carrying a mutation in SOD1 (SOD1-ALS) and two ALS patients carrying a mutation in *TARDBP* (TARDBP-ALS). Blood samples were used to isolate peripheral blood mononuclear (PBMC) on Lymphoprep™ density-gradient centrifugation. The lymphoblastoid cell lines were generated by infecting PBMC with the Epstein–Barr virus as previously described. Cells were grown in suspension in T flasks, in RPMI-1640 medium containing 2 mM L-glutamine, 100 μg/mL streptomycin/penicillin, and 10% (v/v) fetal bovine serum (FBS) and maintained in a humidified 5% CO_2_ incubator at 37 °C. Patients were diagnosed by neurologists from the Hospital Universitario 12 de Octubre (Madrid, Spain) applying El Escorial criteria [39]. Samples were obtained after signing an informed consent form. All procedures were approved by the Hospital Universitario 12 de Octubre and the Spanish Council of Higher Research Institutional Review Board and are in accordance with National and European Union Guidelines.

### 4.2. Compound Preparation

All the compounds were prepared with a stock concentration of 25 or 10 mM in DMSO. The final % of DMSO in cell culture was not higher than 0.1%. BafA1 (Enzo Life Sciences, BCL-CM110-0100, Farmingdale, NY, USA) and MRT68921 (Cayman Chemical, 1190379-70-4, Ann Arbor, MI, USA) were also prepared in DMSO. DFP (Pubchem, 379-405) and hydroxychloroquine (HCQ) were dissolved in water. Carbonyl cyanide-p-trifluoromethoxyphenylhydrazone (FCCP) (Sigma, C2920, St. Louis, MO, USA) was dissolved in ethanol.

### 4.3. Mitophagy Assay

U2OS-iMLS, U2OS-iMLS-PRKN and ARPE-19 MitoQC cells were seeded at a final concentration of 50,000 cells/mL in a 96-well plate (U2OS cells) and in crystals in a 24-well plate (ARPE-19 cells). The next day, cells were treated as indicated. In the case of U2OS-iMLS, 500 μg/mL doxycycline was added to the medium to induce the expression of the reporter. After 24 h, the cells were fixed with 3.7% paraformaldehyde (PFA) 200 mM Hepes and incubated with Hoechst (Invitrogen, H1399, Waltham, MA, USA) to stain the nuclei. The U2OS cells were maintained in the plates in PBS until images were acquired. The ARPE-19 MitoQC cells were seeded in crystals and incubated with DAPI (Sigma-Aldrich, 2D9542, St. Louis, MO, USA) to stain the nuclei. Then, the crystals were mounted over slides with ProLong Diamond Antifade Mountant (Thermo Fisher Scientific, P36961, Waltham, MA, USA). The slides were kept for 24 h, at RT and then at 4 °C until images were acquired.

### 4.4. Immunostaining

The U2OS-iMLS cells were seeded at a final concentration of 50,000 cells/mL in a 24-well plate. The next day, the cells were treated as indicated. After 24 h, the cells were fixed with 3.7% PFA 200 mM Hepes pH 7 and incubated with DAPI (Sigma-Aldrich, 2D9542) to stain the nuclei. The cells were incubated with 0.2% NP-40 to permeabilize them. Then, the cells were washed with 1% BSA in PBS 1X and incubated with the primary antibody (LC3; 1:500, MBL-PM036) for 1h at 37 °C. After washing steps, the cells were incubated with the secondary antibody anti-rabbit Alexa-647 (1:500) for 30 min at RT. Finally, the cells were kept in PBS until imaged.

### 4.5. Image Acquisition

Images of the U2OS-iMLS cells in 96-well plates were obtained under an ImageXpress Micro Confocal microscope (Molecular Devices, San Jose, CA, USA), placed in the Advanced Light Microscopy Facility at Gaustad (University of Oslo, Oslo, Norway). Six images per well were automatically taken at 20X in order to quantify around 1000 cells per condition.

Images of the ARPE-19 MitoQC cells and immunostained U2OS-iMLS cells in 24-well plates were obtained with an AF6000 LX widefield multidimensional microscopy system placed in the confocal laser and multidimensional microscopy in vivo facility at CIB Margarita Salas (Madrid, Spain). Five to six images were manually taken at 40× and to have around 100 cells per coverslip.

Image analysis was performed with CellProfiler [40]. In order to identify red-only structures per cell segmentations of nuclei, cells, and mitochondrial network were performed. Mitochondrial structures were further filtered as “yellow” or “red-only” based on the ratio between their EGFP and mCherry integrated intensities. The final number of red-only structures per cell was used as a mitophagy rate readout. Later, this pipeline was modified in order to analyze mitophagy in cells seeded in 24-well plates. Co-localization between mCherry and LC3 was measured based on the ration between mCherry and LC3 integrated intensities.

### 4.6. Western Blotting

ARPE-19 MitoQC cells were seeded at a final concentration of 200,000 cells/mL in a 6-well plate. After 24 h, the cells were treated as indicated. Proteins were extracted with lysis buffer (50 mM Tris-HCl pH 6.8, 10% glycerol (*v*/*v*) and 2% sodium dodecyl sulfate (*w*/*v*), in distilled water with protease inhibitors 1X (Sigma, P8783) and phosphatase inhibitors (1 mM sodium orthovanadate (Sigma, S6508), 1 mM sodium fluoride (Sigma, 201154), and 5 mM sodium pyrophosphate decahydrate (Sigma, 221368)). The proteins were scrapped and transferred to a tube. The samples were heated at 95 °C for 15 min and spin and stored at −20 °C until used.

Human lymphoblasts were seeded at a final concentration of 10^6^ cells/mL in a 24-well plate and treated as indicated. After washing steps, pellets were stored at −80 °C until used or treated with the lysis buffer to extract the proteins. The samples were heated at 95 °C for 15 min and spun and stored at −20 °C until used.

To quantify protein, a bicinchoninic acid protein assay kit (Thermo Fisher Scientific, 23227, Waltham, MA, USA) was used, and 12 μg of proteins was loaded with 10 mM dithiothreitol and 0.005% bromophenol blue in Criterion TGX Precast Midi Protein gels (BioRad, 5671124, Hercules, CA, USA) and transferred to polyvinylidene fluoride membranes (BioRad, 170−4157, Hercules, CA, USA) activated with 100% methanol (Panreac, 131091.1214, Castellar del Vallès, Spain) for 2 min. The transfer was performed with Trans-Blot Turbo Transfer (BioRad) for 14 min (two waves of 7 min each) at 25 V. After the transfers, protein bands were detected with Ponceau Red (Sigma-Aldrich, 78376, St. Louis, MO, USA). Membranes were washed with PBS 1X-Tween20 (Bio-Rad, 170-6531, Hercules, CA, USA) (PBS-T) and blocked with 5% (*w*/*v*) milk in PBS-T for 1 h, shaking at RT. After blocking, membranes were washed with PBS-T and incubated with primary antibodies anti-LC3 (Sigma L7543, Rabbit, 1:1000), anti-p62 (Abcam 56416, mouse, 1:1000), anti-TOMM20 (Santa Cruz Biotechnology sc-11415, Rabbit, 1:1000, Dallas, TX, USA), anti-TIMM23 (BD Bio 611222, Franklin Lakes, NJ, USA, Mouse, 1:1000), anti-vinculin (Abcam ab129002, Cambridge, UK, Rabbit, 1:1000), and anti-GAPDH (Abcam ab8245, Mouse, 1:1000) in 3% (*w*/*v*) BSA, 0.01% azide in PBS overnight, shaking at 4 °C. Membranes were washed 3 × 10 min with PBS-T before secondary antibody incubation (in blocking buffer for 1 h). The membranes were also washed 3 × 10 min with PBS-T before membrane imaging with the Chemiluminescent system of ChemiDoc™ MP Imaging System from Bio-Rad. Before the reincubation with another antibody, the membranes were consciously washed with PBS-T to remove the previous antibody.

### 4.7. Biochemical Kinase Assay

The inhibition experiments were performed in the MRC Phosphorylation Unit (University of Dundee). ULK1 (5-20 mU diluted in 50 mM Tris pH 7.5, 0.1 mM EGTA, 1 mg/mL BSA, 0.1% mercaptoethanol) was assayed against MBP in a final volume of 25.5 µL containing 50 mM Tris pH 7.5, 0.1 mM EDTA, 10 mM DTT, 0.33 mg/mL substrate, 10 mM magnesium acetate, and 0.02 mM [33P-γ-ATP] (50–1000 cpm/pmol) and incubated for 30 min at room temperature. Assays were stopped by an addition of 5 µL of 0.5 M (3%) orthophosphoric acid and then harvested onto P81 Unifilter plates with a wash buffer of 50 mM orthophosphoric acid.

### 4.8. Animal Samples Analysis

mCherry-GFP-FIS1_101–152_ (MitoQC) male mice with C57BL/6 genetic background [35] were obtained from Dr. Ian Ganley and established in the CIB animal facility. Two animals per group were treated intraperitoneally at P90 (postnatal day 90) with IGS2.7 1 mg/Kg for 8 and 24 h and then sacrificed. The vehicle to treat control animals and to dissolve the drug was composed by 5% Tween-80 and 5% DMSO in NaCl 0.9%.

Prp-hTDP-43(A315T) transgenic male mice (TDP-43^A135T^) and their wild-type (WT) littermate, both with C57BL/6 genetic background, were obtained from Dr. Eva de Lago (Complutense University, Madrid). Four animals per group were treated intraperitoneally with IGS2.7 0.5 and 1 mg/Kg daily for 30 days from P65 until P95. The vehicle to treat control animals and to dissolve the drug was composed by 6.25% Tween-20 and 0.9% DMSO in PBS (Sigma-Aldrich). Animals were sacrificed 24 h after the last drug administration.

B6.Cg-Tg(SOD1-G93A)1Gur/J transgenic male mice (SOD1^G93A^ mice) carrying the G93A mutant form of the human SOD1 transgene (n = 9), and their wild-type (WT) littermate (n = 4), both with C57BL/6 genetic background, were obtained from Dr. Silvia Corrochano (Hospital Clínico). Animals were sacrificed between P112 and P115.

MitoQC spinal cords were extracted and fixed with 3.7% PFA 200 mM Hepes. The rest of the spinal cords were fixed with 4% PFA for 24 h, cryoprotected in 30% sucrose (Merck Millipore, Kenilworth, NJ, USA) and stored at −80 °C. Sections of 20 µm at the lumbar level of the spinal cords were obtained with a cryostat and collected on gelatin-coated slides.

### 4.9. Histological Procedures

Sections of the spinal cords were used for immunofluorescence. Samples were washed with PBS 1X and permeabilized with 0.3% Triton X-100 (Sigma) in PBS 1X for 15 min in a wet chamber. Samples were again washed with PBS 1X and blocked with the blocking solution BGT (3 mg/mL Bovine Serum Albumine (Nzytech, Lisboa, Portugal) 10 mM glycine (VWR Chemicals, Radnor, PA, USA and 0.25% Triton X-100 in PBS 1X) for 1h at RT in a wet chamber. Next, sections were incubated at 4 °C overnight with primary antibodies anti-p26 (Progen, Singapore) and Anti-TOMM20 (Santa Cruz Biotechnology) in blocking solution in a wet chamber. Sections were washed and incubated with secondary antibodies in blocking solution for 1h at RT in a wet chamber. In addition, 1 μg/mL DAPI was used to stain the nuclei. Slides were washed with PBS 1X and mounted with Vectashield antifade mounting medium (Vector Laboratories, H-1000-10, Newark, CA, USA).

### 4.10. Image Acquisition and Analysis

Spinal cords were imaged in the ventral horn area, where the MN boundaries were manually determined based on TOMM20 staining. Images were obtained under CLSMLEICA TCS SP8 STED 3X microscope placed in the confocal laser and multidimensional microscopy in vivo facility at CIB Margarita Salas (Madrid, Spain) with 63x objective. Z-stacks of four to five sections per animal were obtained.

Image analysis was performed with CellProfiler [40]. p62 and TOMM20 area were measured by MN. Ten to fifteen MN were quantified per stack from different Z. First, the mean of the Z-stack (one animal) was calculated. Then the mean of four animals was obtained and represented.

### 4.11. Statistics

Statistics was performed with the software GraphPad Prism 7, which includes the analysis of the data to normal distribution via the Shapiro–Wilk test. The statistical differences between groups were obtained with the unpaired two tailed *t*-test to compare two groups or one-way ANOVA followed by Dunnett’s multiple comparison test if there are more than two groups. A *p*-value lower than 0.05 was considered statistically significant.

## Figures and Tables

**Table 1:** Chemical structures of CK1 inhibitors included in the screening phase, their IC_50_ against CK1 δ/ε, and the percentage of activity of the kinase ULK1.

Compound	Structure	Cellular Assay	CK1δ/ε IC_50_ (nM) [18]	ULK1 %Inhn@10 μM
**IGS2.7**	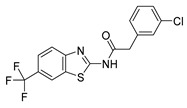	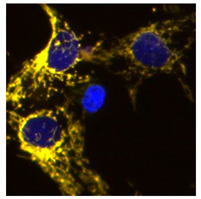	23/840	59 [18]
**IGS3.27**	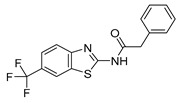	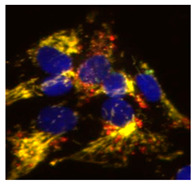	47/750	11.4
**IGS3.4**	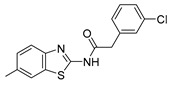	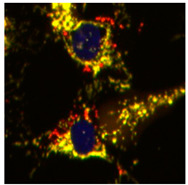	83/880	9

## Data Availability

The datasets supporting the conclusions of this article are available in the DIGITAL.CSIC repository, https://digital.csic.es/handle/10261/36253.
